# A Multi-Resolution Approach for an Automated Fusion of Different Low-Cost 3D Sensors

**DOI:** 10.3390/s140407563

**Published:** 2014-04-24

**Authors:** Jan Dupuis, Stefan Paulus, Jan Behmann, Lutz Plümer, Heiner Kuhlmann

**Affiliations:** Institute of Geodesy and Geoinformation, University of Bonn, Nussallee 17, 53115 Bonn, Germany; E-Mails: paulus@igg.uni-bonn.de (S.P.); behmann@igg.uni-bonn.de (J.B.); pluemer@igg.uni-bonn.de (L.P.); heiner.kuhlmann@uni-bonn.de (H.K.)

**Keywords:** Microsoft Kinect, David laserscanner, automated sensor fusion, markerless registration, surface feature histograms

## Abstract

The 3D acquisition of object structures has become a common technique in many fields of work, e.g., industrial quality management, cultural heritage or crime scene documentation. The requirements on the measuring devices are versatile, because spacious scenes have to be imaged with a high level of detail for selected objects. Thus, the used measuring systems are expensive and require an experienced operator. With the rise of low-cost 3D imaging systems, their integration into the digital documentation process is possible. However, common low-cost sensors have the limitation of a trade-off between range and accuracy, providing either a low resolution of single objects or a limited imaging field. Therefore, the use of multiple sensors is desirable. We show the combined use of two low-cost sensors, the Microsoft Kinect and the David laserscanning system, to achieve low-resolved scans of the whole scene and a high level of detail for selected objects, respectively. Afterwards, the high-resolved David objects are automatically assigned to their corresponding Kinect object by the use of surface feature histograms and SVM-classification. The corresponding objects are fitted using an ICP-implementation to produce a multi-resolution map. The applicability is shown for a fictional crime scene and the reconstruction of a ballistic trajectory.

## 1. Background

In recent years,various optical 3D-sensors have become available and are nowadays used in many different fields of work, e.g., reverse engineering or quality management in industrial tasks, cultural heritage, medicine and also criminal investigations [1]. Several different sensor technologies can be used for 3D digitizing, like terrestrial laser scanners (TLS), triangulation-based range sensors or photogrammetric approaches, like stereo cameras or bundle adjustment of multiple images [1–7]. All of these sensors have their own limitations regarding flexibility, measuring volume, spatial resolution and accuracy. For most applications, it would be sufficient to capture wide areas with an adequate spatial resolution and selected items, like, e.g., statues [5] or evidence objects [8] with a lot more details. Such a demand requires the fusion of different sensors, because a single sensor type is commonly not able to fulfill both requirements. Using low-cost sensors exacerbates this demand. These sensors have more strict limitations regarding measuring volume and resolution.

The advantages and potentials of the combined usage of multiple sensors were presented early on [9] and are used nowadays in a wide range of applications, e.g., the navigation of an unmanned aerial vehicle (UAV) [10], cultural heritage [2–5] and criminal investigation [6–8].

In the field of 3D digitizing, the most frequently used sensor combination consists of a TLS and a digital camera. This combination enables an efficient, flexible and reliable acquisition of large objects, with the advantage of a high spatial resolution and photorealistic representation, and it is mostly used in terms of the documentation of cultural heritage. Many different applications are presented in the literature, where TLS and photogrammetric measurements are used to build a multi-resolution 3D model of well-known cultural sites, e.g., Villa Giovanelli [4] or Pozzoveggiani Church [5], and much more. All of these approaches use manual or semiautomatic algorithms to align point-clouds of different sensors, which are time-consuming and need trained operators.

Similar approaches can also be found in the field of crime scene documentation. An accurate geometrical conservation of crime scenes is of high interest, because the risk of losing evidence by modification by forensic staff, spectators and witnesses is immense. Large scenes have to be acquired quickly and non-invasively, as well as for single evidence objects with high resolution, e.g., for the reconstruction of a ballistic trajectory. Common approaches also use laser scanners in different resolution settings [1] or stereo camera systems [11] to produce multi-resolution models with a 3D accuracy of approximate 3 mm in the case of TLS [12].

One approach in the field of crime scene documentation used the optical digitizer “Konica-Minolta Vivid 910” in different sensor configurations to generate a multi-resolution map [8]. This triangulation-based range sensor works within a range between 0.6 m and 2.5 m with an accuracy up to a tenth of a millimeter [13] and can be equipped with different optics (wide, middle, tele) to acquire point-clouds in different resolution classes. Using these optics, a simulated crime scene was imaged from multiple viewpoints, and various point clouds with different resolutions were recorded. These point-clouds were aligned using the semiautomatic procedure “ImAlign” from the commercial PolyWorks software.

Nevertheless, most of the approaches required the interaction of an operator for the alignment of single 3D point-clouds or the fusion of 3D range and 2D image data.

Locating and aligning 3D-models to a scene containing multiple different objects is a well-known problem in computer vision. So-called 3D keypoint detectors [14,15] are used to generate and describe a set of distinct points of the model and all points of the scene. Thus, homologous keypoints of the model and the scene can be used to calculate the 3D-transformation matrix between both datasets.

A further application where automated object recognition is required is the well-known “bin-picking problem” in robotics. The goal of the bin-picking approach is the automated interaction of a robot with its direct environment. Therefore, objects that should be picked up by a robot have to be identified in a 2D or 3D image. The recognition step is commonly realized by using different feature descriptors, like, e.g., scale-invariant feature transform (SIFT) [16] or fast directional chamfer matching (FDCM) [17] in the 2D case and, e.g., the RANSAM (random sample matching) algorithm [18] in the 3D case.

Using established commercial sensors for data acquisition is quite expensive and requires intensive operator training. With the rise of different low-cost 3D sensors, an economic and simple imaging alternative is available. These sensors usually work within the same accuracy range as the more expensive ones, but with the advantage of much lower investment costs (Table 1) [19]. Furthermore, the technology of low-cost systems, which are based on consumer products, is commonly very user-friendly; thus, they are usable without intensive training.

In this study, we present an automated alignment approach for 3D point-clouds. This approach combines the advantages of multiple sensors regarding measuring volume and resolution by generating a multi-resolution map. We focus on the fusion of unorganized point-clouds coming from different sensor using a characteristic 3D shape description, the so-called “Surface Feature Histograms” [20], in combination with a support vector machine (SVM) classification [21]. These histograms have shown their applicability for the alignment of different viewpoints for low-resolution laser scans [22,23], as well as for the detection of different geometrically distinct objects with high resolution [20].

Same objects coming from the two different sensors are automatically assigned and finally aligned using established procedures, like the iterative-closest-point (ICP) algorithm. Thus, our method can be used as a generalized approach for the generation of multi-resolution maps of various applications using an automated assignment based on a classification with surface feature histograms.

To verify the applicability of the approach, different fictional crime scenarios are measured and processed with the presented algorithm. The Microsoft Kinect sensor [24] and the David laser scanning system [25] are used as low-cost 3D scanning devices.

## 2. Method

### 2.1. Data Acquisition

In the experiment, the signals of two different low-cost sensors are separately recorded and subsequently combined. The first sensor is the Kinect from Microsoft company, a structured light sensor developed for gesture and pose determination in video games. Here, it is used for the acquisition of the original scenarios, resulting in a low-resolved, wide-range point-cloud. The second sensor is the David laser scanning system, which is used for precise and highly detailed scans of single objects. Because the David system provides a higher resolution compared to the Kinect sensor, the David system is defined as the “high-resolved” or “high-resolution” sensor and the Kinect as the “low resolved” or “low-resolution” sensor. A comparison of both sensors is shown in Table 1.

#### 2.1.1. Microsoft Kinect

**Figure 1.**
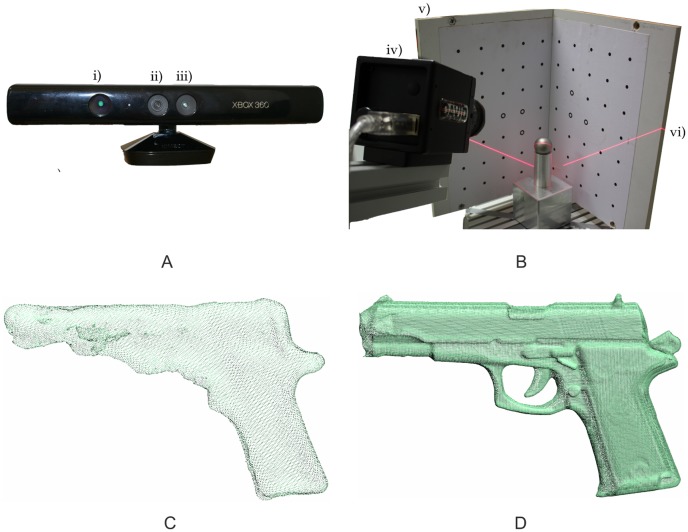
The low-cost sensors, Kinect (**A**) with the IR emitter (i), the RGB camera (ii) and the IR camera (iii), as well as the David system (**B**) with the camera (iv), the calibration corner (v) and the laser line (vi). Corresponding point-clouds (**C,D**) from the Kinect sensor and the David system, respectively.

The Kinect sensor, designed for natural interaction in computer gaming environments [26], combines an infrared projector, an infrared camera and an RGB camera (Figure 1A). It captures a depth image with 640 × 480 pixels by a structured light approach. For the projection, an infrared laser beam is split into a defined pattern, which is recorded by the infrared camera. By comparison to a previously calibrated reference pattern, a disparity image is calculated [27]. Factory-supplied calibration parameters enable the extraction of a point-wise distance value as the distance between the sensor and the object by the principle of triangulation.

The accuracy of this depth image has been investigated by several groups and is mainly influenced by the distance between the sensor and the object [27]. The Kinect is capable of measuring objects within a distance of 0.5 to 5 m. With an increasing distance, the accuracy decreases from a standard deviation (SD) of a few millimeters to about 4 cm [26], and the point to point distance increases from 0.9 mm to 7 mm [24,27].

The high measuring frequency of 30 frames per second produces a huge amount of redundant information if rigid objects are observed, which can be used for more accurate and complete 3D models [27]. The program used for the generation of a meshed 3D point-cloud from the depth video stream picks up the well-known KinectFusion algorithm [28] and is available as ReconstructMe console version 0.6.0–405 [29].

To reconstruct a meshed 3D point-cloud, the KinectFusion algorithm uses an ICP algorithm to track the 6 degrees of freedom pose of the sensor. The movement of the sensor can be realized in two different ways. One possible procedure is the movement of the Kinect around the objects under testing. In this case, the pose of the sensor changes in an arbitrary way; thus, 3D reconstruction is less accurate. Another way is to simulate sensor movement by a rotation of the objects. This can be realized by using a rotary table and a fixed position of the Kinect sensor. The advantage of this strategy is the constant change of the 6 degrees of freedom. Thus, the tracking of the sensor is more accurate, which results in a more accurate point-cloud.

Summing up, the Kinect is able to capture scenes to a suitable extent, but its low resolution of 0.5–4 mm prevents the recording of small details (Figure 1C).

#### 2.1.2. David Laser Scanning System

The David laser scanning system is developed for the precise point-cloud acquisition of single objects. In its basic configuration, the David system only requires a USB-attached digital camera, e.g., a webcam, a hand-held laser emitter with line optics and a so-called “calibration corner” with a calibration pattern specified for the David software (*cf.* Figure 1B). The David system basically works according to the “light-section method” [30], which is comparable to the laser-triangulation technique used in commercial setups [20]. However, because of the hand-held laser setup, the point estimation process is slightly different, as presented in [31]. The David software estimates the relative orientation of the camera with respect to the calibration corner. During the measurement process, the software uses points of the laser line lying on the two planes of the calibration corner to reconstruct the laser plane. Based on the estimated imaging geometry, the 3D-object-points are calculated from the contour of the laser line on the CCD-chip. Therefore, the center of gravity (CoG) of the received laser line is calculated column by column.

Non-published investigations at the university of Bonn pointed out that the measurement accuracy of the David laser scanning system is closely linked to the imaging geometry, primarily regarding the incident angle between the laser plane and the calibration corner. Thereby, the smallest uncertainties were obtained at an incident angle between 35°and 45°. Using a classical David setup leads to variations of the imaging geometry, due to the hand-held laser. To achieve the highest accuracy and reliable scanning results, all David components are integrated in the measuring setup (Figure 1B), including a motorized laser guidance and a rotary plate for multiple viewpoints. The object under test is placed in the rotational axis of the rotary plate, and the laser line is moved over the object's surface with constant speed and an invariant imaging geometry. Afterwards, the viewpoint is changed by rotating the object by a defined angle. These steps are repeated until the whole surface is measured. All single point-clouds are aligned automatically and fused using the software-internal algorithm supported by the predefined rotation angles. Thus, it is possible to align the point-clouds of rotationally symmetric objects.

Summing up, the David system is able to capture small objects with a high resolution (Figure 1D), but in a severely limited measuring range of several decimeters.

### 2.2. Algorithm

**Figure 2.**
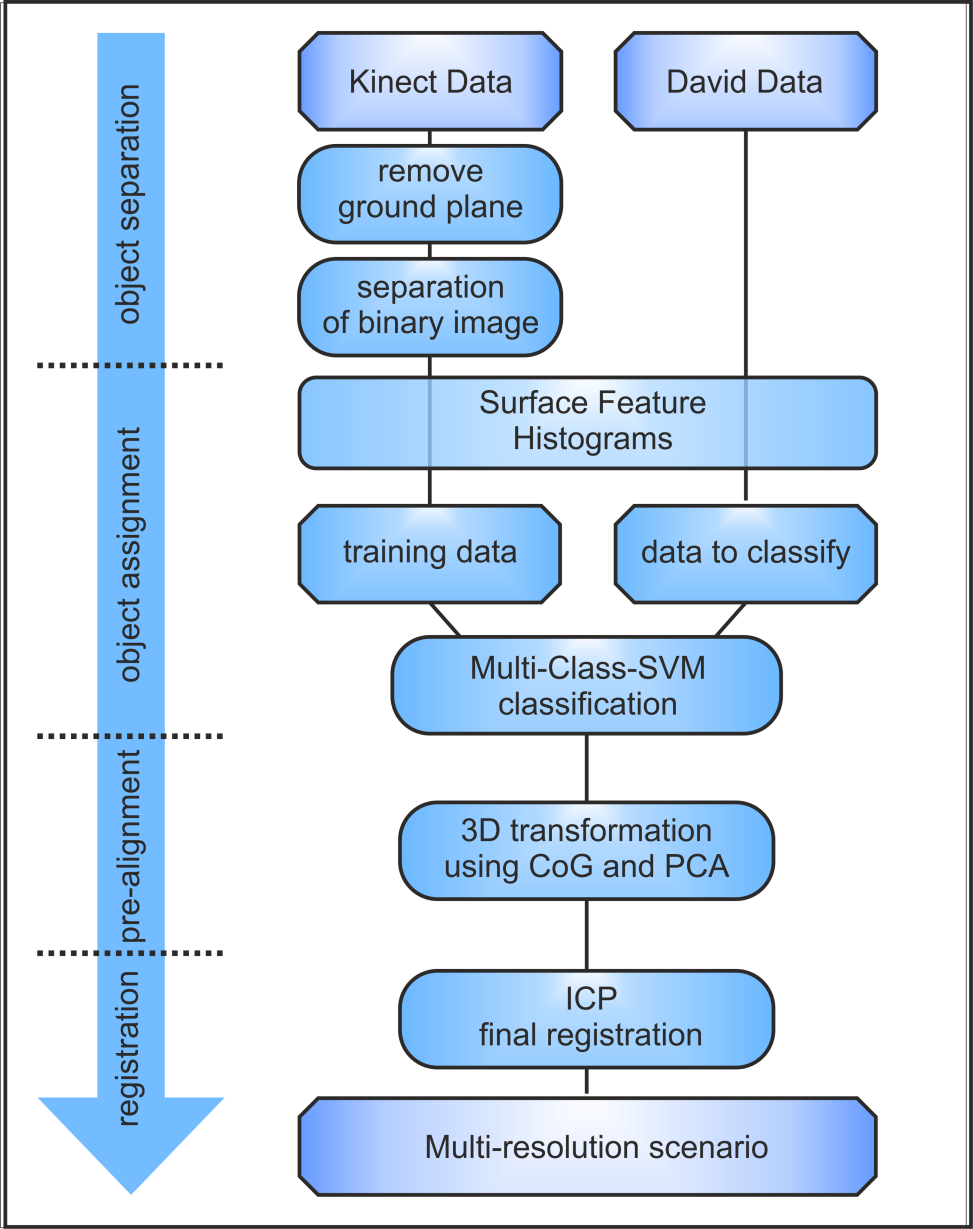
Dataflow chart that describes the main steps of the automated registration approach. CoG, center of gravity.

In this study, an algorithm for the automated assignment and alignment of highly resolved David point-clouds of different objects to a low-resolved Kinect point-cloud, including several objects, is presented. Therefore, four different steps are necessary to obtain a complete transformation matrix. The general dataflow is presented in Figure 2.

#### 2.2.1. Object Separation of Kinect's Point-Cloud

**Figure 3.**
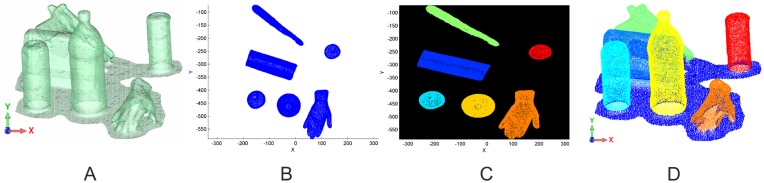
Results of the segmentation work-flow: (**A**) original point-cloud; (**B**) the ground-plane was removed and the point-cloud was rotated into the xy-plane; (**C**) segmented binary image; (**D**) segmented point-cloud retransformed into the origin space.

In the first step, the imaged objects have to be extracted from the Kinect point-cloud. The approach used for the separation is based on the detection of connected components in a binary image representing the presence of an object. Several tasks are necessary to generate a binary 2D image from a 3D point-cloud. In this image, pixels denoting an object occurrence are separated from those without. Regarding the original scene in Figure 3A, first of all, the ground-plane of the imaged scene has to be removed. Using a RANSAC-algorithm, the main plane in the point-cloud is determined, and all points within a fixed range to plane are removed. The remaining point-cloud is rotated in a way that the normal vector of the ground-plane is perpendicular to the xy-plane (Figure 3B). The transformed points are projected onto the xy-plane, rasterized and converted into a binary image (Figure 3C). Connected components in the binary image correspond to single objects. These objects are derived from the image and linked to the original point-cloud (Figure 3D).

#### 2.2.2. Assignment of David and Kinect Objects

**Figure 4.**
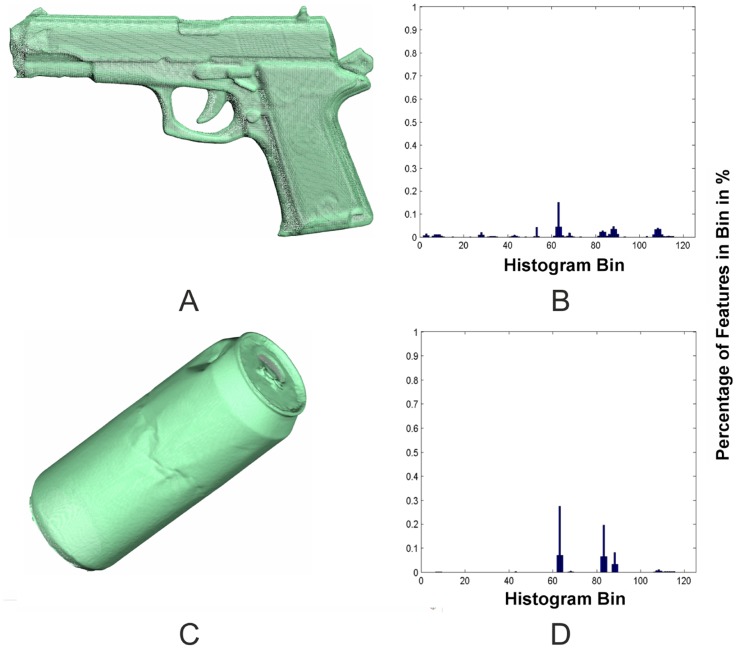
Distinct surfaces generate distinct histogram manifestations. A pistol (**A**) with the corresponding mean histogram (**B**) is shown, as well as a can imitation (**C**) with the mean histogram (**D**).

In a second step, the highly resolved David scans have to be assigned to their corresponding Kinect objects. Because the objects only consist of unsorted 3D-points and no semantic information is available, a representation of these objects is needed that enables a clear assignment. Because the David system and the Kinect produce point-clouds of different resolutions in different coordinate systems, the representation has to be invariant with regard to density and pose. One approach to satisfy this requirement is the use of surface feature histograms. Developed by [22,23] for the demands of robotics and adjusted by [20] for highly resolved laser scans, these histograms provide a clear representation of the surface geometry based on differential geometric features. The basic idea of these histograms is that 3D-points lying on surfaces of different geometric primitives have a characteristic point distribution within a local neighborhood. This distribution is represented by the histograms (Figure 4), which are independent of pose, resolution or level of noise. The histograms encode the differential geometric relation between the normal vectors in a local neighborhood, calculated out of small regions of the object's surface. Following this approach, the normal vectors for every 3D-point have to be calculated from a defined neighborhood using principle component analysis (PCA). Surface feature histograms use 3D points and their normal vectors within a small neighborhood to calculate a representation of the surface geometry, encoded in a histogram [20]. The result is a multitude of histograms for every object, containing histograms of all the geometries that are included in the object. Transferred to the actual registration problem, surface feature histograms can be used to find correspondence between Kinect and David objects.

To assign Kinect and David scans, the distribution of the histograms of every single Kinect object has to be compared to the histogram distribution of the David objects. A straight-forward approach to solve the assignment task would be the evaluation of distance functions between the histograms. However, we used a one-*vs.*-one non-linear multi-class classification by SVM (support vector machine), implemented in LIBSVM [32], because of the convincing performance facing the high dimension and high amount of the histograms [20].

The histograms from the Kinect objects are used to learn a classification model that classifies the histograms of the David objects. Each 3D point in the David point-clouds is assigned to one Kinect object. The Kinect object, having the maximum amount of assigned points, is selected as the corresponding object.

If objects are represented by similar geometric primitives, the classification could assign multiple David objects to a single Kinect object. In this case, the assignments with the highest percentage of supporting inliers is selected. The other David object, which is also assigned to the Kinect object, but which has a lower support, is assigned to the Kinect object that has the second largest support. This procedure is repeated recursively, until there are only unique assignments left.

#### 2.2.3. Pre-Alignment

The assignment step is followed by a pre-alignment of corresponding objects. To obtain good initial values for the final ICP registration, a rough transformation is performed using the CoG and PCA. In detail, the translation vector is calculated from the difference of the CoGs and the rotation is derived from the principle components. While the calculation of the translation is unambiguous, the rotation is ambiguous, as the direction of the principle components of both the Kinect and the David object are uncertain. For this reason, all possible combinations of the main axis orientations are used, and the one having the smallest deviations, defined by the average nearest neighbor distance, is used to calculate the initial rotation for the final ICP registration.

#### 2.2.4. Final ICP Registration

In the last step, the relative orientation of the assigned and pre-aligned David and Kinect objects can be optimized using the ICP algorithm. As presented in Section 2.1, objects within the original crime scene are scanned using the Kinect sensor and picked up for detail scans by the David measuring system. For this reason, the point-clouds are acquired from different viewpoints. Measurements performed by the David sensor may provide scans of parts that are not visible in the Kinect scan. The bigger the amount of missing points, the bigger will be the amount of bad assignments in the ICP algorithm. Therefore, the points without counterparts in the other model should be excluded from ICP. In the presented method, the worst 10% of the points were excluded at each object, because the automatic detection of interfering points and the object-specific adaption of an exclusion percentage is not possible.

## 3. Results

In this section, the results of the presented registration approach applied to point-clouds of a fictional crime scene are illustrated. The scenario includes possible crime scene objects, like a pistol, a cut body part (plastic material) and some typical household items. To achieve a high accuracy, all the objects were arranged on a rotary plane and scanned from a fixed Kinect position. In a second step, these objects were picked up and scanned using the David laser scanning system to archive a highly resolved point-cloud. To minimize measurement uncertainties caused by specular reflections at the object's surface, objects, like the can or cups, were coated with diffuse reflecting scan spray [25], distributed by David company. This is acceptable, since the coating of non-natural surfaces does not disturb the objects irreversibly and is comparable to the procedures used for fingerprint identification.

### 3.1. Object Assignment and Pre-Alignment

**Figure 5.**
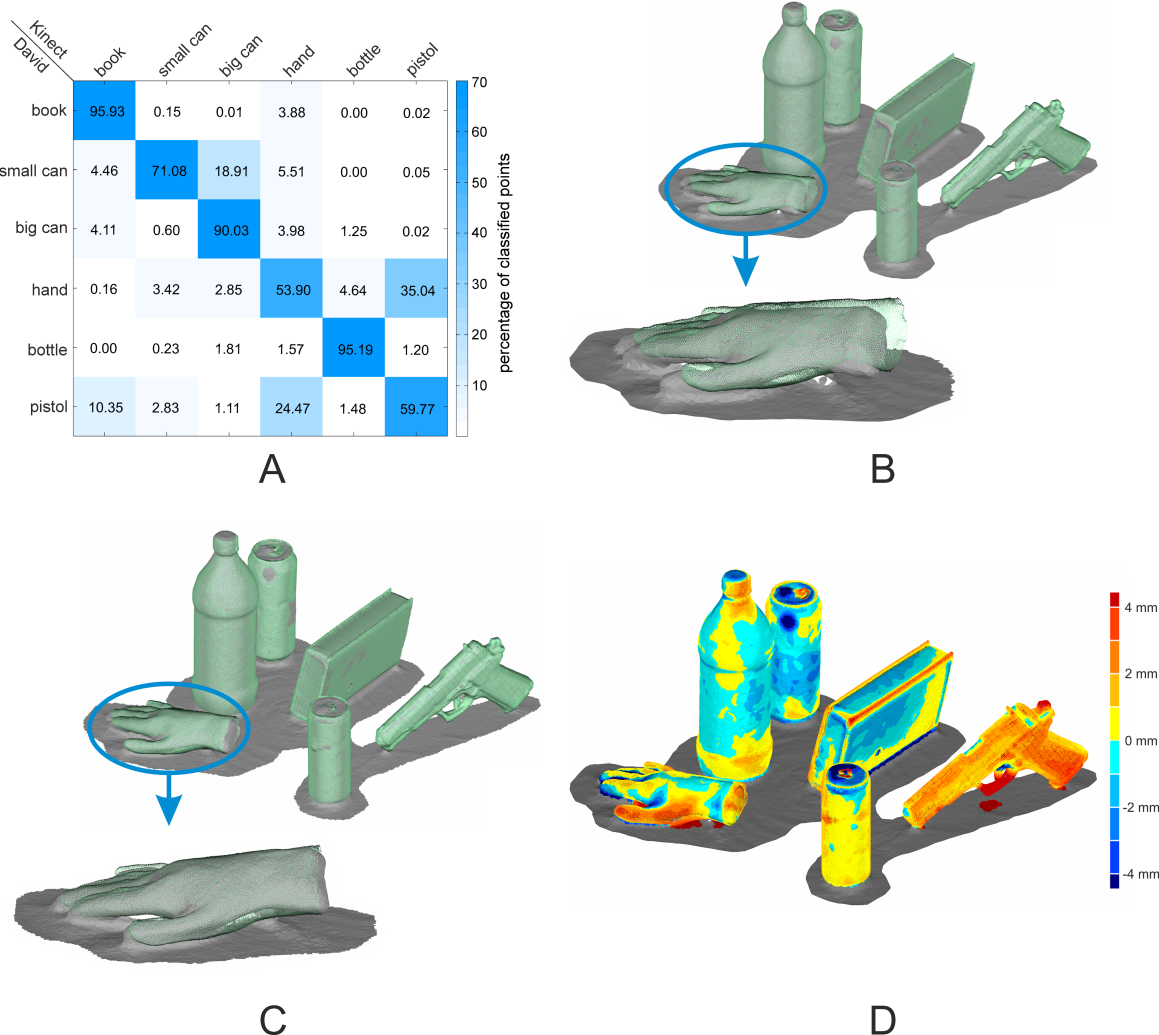
(**A**) The results of the object assignment using SVM classification; (**B**) point-clouds of the pre-aligned objects; (**C**) point-clouds after final ICP registration; (**D**) deviation map of registered point-clouds.

The David and the Kinect objects were assigned using a multi-class SVM classification on the histogram level. This approach results in a percentage of inliers for every object combination and allows a reliable assignment (Figure 5A). The experimental results underline that surface feature histograms are well suited for the identifications of objects in scenes obtained by different sensors. Regarding the main diagonal in Figure 5A, all David objects were correctly assigned to their corresponding Kinect object with high certainty. A remarkable result was obtained for the two cans and the bottle. These objects are representable by cylindrical primitives that differ only in the radii. Nevertheless, the surface feature histograms combined with a multi-class SVM were able to differentiate these objects clearly with a percentage of inliers ranging from 71% to 95%. For the pre-alignment, the CoG and the main axes were used. Concerning the results in Figure 5B, a small offset into the direction of the ground plane's normal vector between the objects remains. Nevertheless, the pre-alignment is sufficient to be used as the initial solution for the final ICP registration.

### 3.2. ICP Registration

The resulting point-clouds of the ICP registration are illustrated in Figure 5C,D. As the quality of the pre-alignment is sufficient, the ICP compensates for the remaining deviations of the point-clouds of the two sensors. The most positive improvement can be found in the case of the hand, where the offset is completely compensated.

Because there are no ground-truth data available, the only measure of precision is the root mean square error (RMSE) value. This error value is derived from the Euclidean distance between corresponding point pairs in both point-clouds, used for the ICP registration. The RMSE for the presented scene ranges from 1.5 mm in the case of the bottle to 3.9 mm in the case of the pistol. Regarding the low resolution and the poor ability of the Kinect sensor to resolve small details, the deviations are obtained from the difference of the sensors' point-clouds.

### 3.3. Practical Application

A practical application in forensic analysis is the reconstruction of a ballistic trajectory [1]. For this application, two criteria have to be satisfied. On the one hand, the position of the involved objects within the scene has to be determined. On the other hand, small bullet holes have to be detectable in the involved objects.

**Figure 6.**
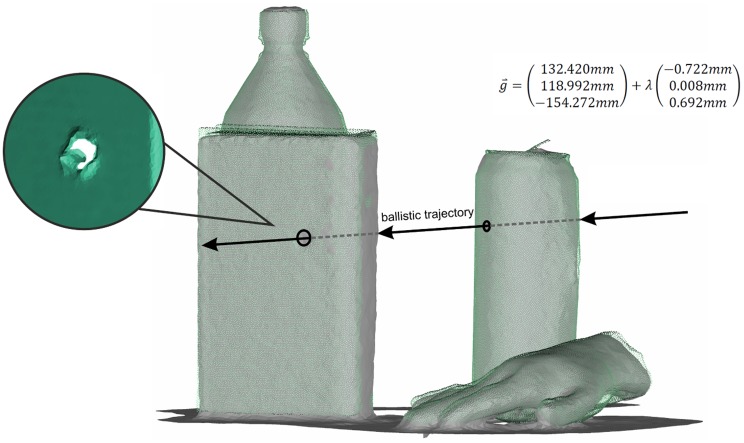
Result of a reconstruction of a ballistic trajectory.

Both requirements are fulfilled using the presented approach combining the Kinect sensor and David laser scanning system. Therein, the high-resolved David scans enable the identification of small details in the point-cloud, like bullet holes, while the Kinect sensor images the whole crime scene with low resolution to acquire the spatial position of the objects. Figure 6 shows a fictional crime scene where the can and the book were primed with small holes representing the bullet holes. It becomes clear that the holes are only detectable in the David point-cloud, due to the low resolution of the Kinect sensor. For a parametric representation of the ballistic trajectory, the centers of the bullet holes were estimated on the aligned David point-cloud using the point feature extraction algorithm of Geomagic Studio 12. Afterwards, the final ballistic trajectory is reconstructed by an estimation of a line feature connecting the center points of the bullet holes.

### 3.4. Further Results

To verify the general applicability of the presented approach, different fictional scenes containing various household and crime scene items were scanned by both sensors and fused automatically. Table 2 shows the results of the tested objects regarding the RMSE for every object averaged over several scenes.

The tested scenes resulted in an averaged RMSE between 1.46 mm and 5.39 mm, with a maximum range between 1.43 mm and 8.65 mm. The largest averaged RMSE was obtained from the book object. It ranges from 1.88 mm to 8.65 mm with a maximum RMSE that is more than two times higher compared to the other objects. The reason for this is the position of the book in the original scene. If the book was lying on its cover, the bottom side was not imaged by the Kinect sensor in contrast to the David system, resulting in a misallocation of corresponding points in the ICP algorithm and, therefore, a higher RMSE.

## 4. Discussion

We presented an approach for the automated fusion of low-resolution and high-resolution point-cloud data. This was evaluated by using the Microsoft Kinect as a low-resolution sensor and the David as a high-resolution scanning system. The resulting multi-resolution maps provide the representation of big scenes with single items scanned in detail. The main contribution of our work is a novel solution to the problem of point-cloud registration using a classification approach based on geometrical features. The histogram-based assignment operates reliably for objects coming from the two different sensors with different resolution and accuracy classes. The presented scenes included, in addition to other geometries, some household objects, like a bottle or cans, which are all representable by cylindrical geometric primitives. However, our approach uses small changes in the histogram characteristic to assign objects of the same type reliably. Despite the good results reached when using a fictional crime scene scenario, some limitations have to be discussed.

For an appropriate segmentation of the Kinect point-cloud, some limitations have to be taken into account. The subsurface has to be representable by a plane, as it is identified by a RANSAC and removed afterward. Highly curved surfaces or different levels of height may result in an unusable separation of the point-cloud. A further restriction concerns the position of the objects. The presented approach uses connected components on a binary image generated from the projection of the transformed point-cloud on the xy-plane, and therefore, single objects are not allowed to touch one other. In case of two or more tangent objects, the connected components algorithm would only detect one object, including the points of several ones. More advanced approaches for point cloud segmentation may overcome this limitation, but were not evaluated during this study [23,33].

The pre-alignment step using the CoG and the main axes of the objects gives a good initial transformation. Nevertheless, a small offset between both point-clouds was found. This offset is caused by the different viewpoints of the sensors. While the Kinect sensor images the scene in the original arrangement, objects were picked up and moved to the David laser scanning system, where they are imaged from different viewpoints. For this reason, object parts, like the bottom of the cut hand, were only imaged by David, with the result that the CoGs of the point-clouds were slightly different. As a consequence, the pre-aligned David point-clouds can only act as an initial solution for ICP registration. A further restriction concerns rotary-symmetric objects. These objects depict a general problem when using automated alignment approaches.

Regarding the approaches in the field of cultural heritage and crime scene documentation, where the sensor fusion and the registration of point-clouds were commonly performed interactively [4] or semi-automatically [8], our approach has the advantage of a completely automated object assignment and registration. Excepting the limitations in the segmentation step, the assignment and alignment are basically transferable to any type of 3D digitizing sensors.

In comparison with the bin-picking approaches, where only the position of the objects in the robot's coordinate system is derived, our classification additionally enables the generation of semantic information, like the attribution of geometric primitives to every data point. In Paulus *et al.* [20], surface feature histograms were successfully used to separate 3D point-clouds of plants into leaf and stem points. Thus, the histograms could be used to locate specific objects of one special type.

With the usage of two low-cost sensors combined in an automatic fusion algorithm, the costs for sensors, software and operators can be reduced significantly, compared to commercial sensors, like the Vivid 910 [8] or TLS [5].

As presented in Table 3 the point-cloud resolution and the accuracy of the used low-cost sensors is definitely comparable to those of more expensive ones [19]. Furthermore, these sensors are quite fast and easy to use, what reduces the effort for data acquisition as well as the cost and time for operator training. Because of the continuous hand-operated measuring process of the Kinect sensor, there is no need for multiple stations and the measuring volume is principally unlimited with respect to the memory space of the operating system [34].

To avoid the limitations in the object segmentation and pre-alignment steps, the objects' texture can be included in the algorithm. The latest version of ReconstructMe [29] software, as well as the David software, enables the acquisition of the surface texture, which may support the object segmentation and the pre-alignment. One approach in this context was presented by [35] for the registration of depth images obtained from depth cameras. They combined the ICP and the SIFT algorithm to an RGBD-ICP and successfully applied it to different scenarios. A similar approach would be conceivable in future studies.

An advancement that will be addressed in our further research concerns the David laser scanning system. In its current configuration, the objects have to be picked up and placed inside the calibration corner. However, using a extended David setup, as presented in [36], detailed object scans can be performed locally without any changes of the original scene arrangement. This strategy would additionally solve most of the limitations in the pre-alignment step, because the objects were scanned from the same viewpoint. However, the sensor fusion is still necessary, because the field of view of the mobile David system is still limited.

## 5. Conclusions

We introduce surface feature histograms as features for a classification that detects identical objects in point-clouds with different properties. By the use of two sensors with different resolution classes, a detailed 3D-representation of single objects is possible without losing the original spatial arrangement. The object's position is given by the low-resolution Kinect scan, while a more detailed representation is given by the David system. A fully automated fusion of the point-clouds with different resolutions was provided by the use of surface feature histograms and the ICP algorithm. This approach enabled the generation of a multi-resolution map for the documentation of arbitrary scenes, where a low-resolution map is enriched with high-resolution scans of interesting objects.

## Figures and Tables

**Table 1.** Overview of the main sensor properties for the David and the Kinect sensor.

	**Microsoft Kinect**	**David laserscanner**
cost	∼100 €	∼1,000 €
resolution	∼0.2% of the object size	depending on the measuring setup
accuracy	depending on object fragmentation	∼0.1% of the object size
used wavelength	827 nm	660 nm
measurable volume	distance of 0.5 m to 5 m	depending on the measuring setup
manual registration	no	yes

**Table 2.** Point-cloud sizes and RMSE values of the objects used (*k* = ×1, 000).

**Object**	**# points Kinect**	**# points David**	**averaged RMSE (mm)**	**min RMSE (mm)**	**max RMSE (mm)**
book	38 k	292 k	5.39	1.88	8.65
big can	36 k	406 k	1.84	1.67	2.15
small can	28 k	228 k	1.80	1.73	1.91
plastic hand	25 k	594 k	3.46	3.37	3.65
pistol	32 k	237 k	3.77	3.55	4.02
big cup	36 k	626 k	2.34	2.10	2.58
small cup	24 k	320 k	3.12	2.93	3.42
bottle	59 k	144 k	1.46	1.43	1.49

**Table 3.** Comparison of the classical terrestrial laser scanners (TLS) approaches to our method.

	**TLS Approach**	**Our Approach**
price in €	>30,000	<1,500
measuring volume	up to 130 m	a few meters
spatial accuracy	up to 3 mm	∼3 mm
detailed accuracy	up to 3 mm	up to 0.2 mm
data processing	manual/semiautomated	automated
commercial post-processing software	required	not required
